# Clinical characteristics and outcomes of patients with end-stage renal disease hospitalized with diabetes ketoacidosis

**DOI:** 10.1136/bmjdrc-2019-000763

**Published:** 2020-02-27

**Authors:** Rodolfo J Galindo, Francisco J Pasquel, Maya Fayfman, Katerina Tsegka, Neil Dhruv, Saumeth Cardona, Heqiong Wang, Priyathama Vellanki, Guillermo E Umpierrez

**Affiliations:** 1 Division of Endocrinology, Emory University School of Medicine, Atlanta, Georgia, USA; 2 Rollins School of Public Health, Emory University, Atlanta, Georgia, USA

**Keywords:** ketoacidosis, dialysis, ESRD, hypoglycemia

## Abstract

**Introduction:**

There is limited evidence to guide management in patients with end-stage renal disease (ESRD) on chronic hemodialysis admitted with diabetes ketoacidosis. Thus, we investigated the clinical characteristics and outcomes of patients with ESRD admitted with diabetic ketoacidosis (DKA).

**Methods:**

In this observational study, we used International Classification of Diseases Ninth/Tenth Revision codes to identify adult (aged 18–80 years) patients admitted to Emory University Hospitals between 1 January 2006 and 31 December 2016. DKA and ESRD diagnoses were confirmed by reviewing medical records and by admission laboratory results.

**Results:**

Among 307 patients with DKA meeting the inclusion and exclusion criteria, 22.1% (n: 68) had ESRD on hemodialysis and 77.9% (n: 239) had preserved renal function (estimated glomerular filtration rate >60 mL/min/1.73 m^2^). Compared with patients with preserved renal function, the admission blood glucose was higher (804.5±362.6 mg/dL vs 472.5±137.7 mg/dL) and the mean hemoglobin A1c was lower (9.6%±2.1 vs 12.0%±2.5) in patients with DKA and ESRD, both p<0.001. The rates of hypoglycemia <70 mg/dL (34% vs 14%, p=0.002) and <54 mg/dL (13% vs 5%, p=0.04) were higher in the ESRD group. During hospitalization, more patients with ESRD develop volume overload (28% vs 3%, p<0.001) and require mechanical ventilation (24% vs 3%, p=<0.001). There were no differences in hospital mortality (3% vs 0%, p=0.21), but length of stay (median 7.0 vs 3.0 days, p<0.001) was longer in the ESRD cohort. After adjusting for multiple covariates, patients with DKA and ESRD have higher odds of hypoglycemia (OR 3.3, 95% CI 1.51 to 7.21, p=0.003) and volume overload (OR 4.22, 95% CI 1.37 to 13.05, p=0.01) compared with patients with DKA with preserved renal function.

**Conclusions:**

Patients with DKA and ESRD on chronic hemodialysis had worse clinical outcomes including higher rates of hypoglycemia, volume overload, need for mechanical ventilation and longer length of stay, compared with patients with preserved kidney function.

**Significance of this study**

**What is already known about this subject?**

- There is limited evidence on the management and outcomes of patients with end-stage renal disease (ESRD) admitted with diabetic ketoacidosis (DKA).

**What are the new findings?**

- Compared with patients admitted with DKA and preserved renal function, patients with ESRD (1) presented with higher admission blood glucose, but lower hemoglobin A1c; (2) had higher rates of hypoglycemia, volume overload and need for mechanical ventilation; and (3) have similar mortality, but longer length of stay and higher hospital costs.

**How might these results change the focus of research or clinical practice?**

- The poor clinical outcome observed in patients with ESRD highlights the need for close glucose monitoring and a personalized approach to volume replacement during treatment for DKA; as well as the need for prospective studies to assess personalized management algorithms to improve treatment-related complications and reduce healthcare utilization in this high-risk population.

## Introduction

In 2015, there were almost 700 000 patients with end-stage renal disease (ESRD) in the USA.^1 2^ Diabetes is a leading cause of chronic kidney disease and ESRD, with approximately 40%–50% of patients on dialysis having a diagnosis of diabetes.^1–3^ The adjusted survival after 3 and 5 years of hemodialysis initiation is 55% and 40%, with patients with diabetes having the worst adjusted survival rates.^2–4^

Diabetic ketoacidosis (DKA)^5^ is a serious and life-threatening hyperglycemic emergency that occurs in patients with type 1 and type 2 diabetes.^6 7^ In a nationwide study in the USA, we recently reported that up to 7.5% of patients admitted with DKA had ESRD listed as a comorbidity.^6^ The diagnosis and management of DKA in patients with ESRD is challenging due to alterations in glucose metabolism, insulin sensitivity and altered renal clearance of antidiabetic medications.^3 8^ Despite extensive data from landmark studies on the management of DKA in general populations,^6 9^ there is virtually no evidence to guide appropriate volume replacement, insulin and potassium therapy in patients with DKA and ESRD.^10^ We recently reported that ESRD in patients with DKA was associated with higher adjusted risk for all-cause readmissions, with most readmissions occurring within 2 weeks after discharge.^6^

A major limitation of prior studies has been the use of International Classification of Diseases Ninth/Tenth Revision (ICD-9/10) codes to identify the diagnosis of DKA, without confirmation of the diagnosis based on admission biochemical/laboratory data.^6 9^ Accordingly, we performed a detailed analysis of clinical and laboratory characteristics to confirm the presence of DKA and clinical outcomes in patients with ESRD on hemodialysis admitted to our tertiary referral academic center. We hypothesized that patients with ESRD, presenting with DKA, will have increased morbidity and treatment-related complications compared with patients with DKA and preserved kidney function.

## Methods

### Study population

We included adult patients between 18 and 80 years of age, with admission ICD-9/10 codes for DKA and ESRD on chronic maintenance hemodialysis, hospitalized at Emory University Hospitals between 1 January 2006 and 31 December 2016. We excluded pregnant patients, those not meeting the laboratory diagnosis.^11^

### Cohort creation

This retrospective study searched admission data via the Emory Clinical Data Warehouse. After identifying patients with admission diagnosis of DKA and ESRD on hemodialysis by prespecified ICD-9/10 criteria (see online supplementary materials), we included only the first admission. We then confirmed the diagnosis of DKA for all subjects by using prespecified laboratory diagnostic criteria set by the American Diabetes Association (ADA; see online supplementary materials).^11^ The diagnosis of ESRD was confirmed by laboratory data (estimated glomerular filtration rate (eGFR) <15 mL/min/1.73 m^2^) on admission. For comparison, we included patients with DKA and preserved renal function with eGFR >60 mL/min/1.73 m^2^.

10.1136/bmjdrc-2019-000763.supp1 Supplementary data

### Study variables

We collected patient’s demographics: age, gender, race, ethnicity, body mass index, admission, and inpatient laboratory values including hemoglobin A1c (HbA1c), glucose, pH, ketones, total serum osmolality (2(Na)+18/glucose+blood urea nitrogen/2), anion gap, serum sodium, serum potassium, and eGFR.

Comorbidities and hospital complications were identified by ICD-9/10 codes generated during the hospitalization as previously described.^12^ ICD-9 codes generated during the hospital stay, but not present during admission, and laboratory data generated during the stay were used to calculate the rate of hospital-related complications, including: hypokalemia (<3 mmol/L), severe hypokalemia (<2.5 mmol/L), myocardial infarction, volume overload (pulmonary edema or congestive heart failure), need for mechanical ventilation, deep vein thrombosis, pulmonary thromboembolism, sepsis, and rhabdomyolysis. We report all-cause mortality during hospitalization, and following 30 and 365 days after discharge. We also analyzed length of hospital stay (LOS) and hospitalization costs. Response to treatment was analyzed as time to normalization of glucose (blood glucose (BG) <250 mg/dL) during treatment, rates of hypoglycemia <70 mg/dL, <54 mg/dL and <40 mg/dL, rates of hypokalemia, mean daily glucose, etc. BG values included point-of-care testing and laboratory glucose measures. Patients were managed by their primary care teams with a nurse-driven protocol available across all Emory Hospitals, based on the ADA guidelines for DKA management.

### Statistical analysis

Major outcomes of interest included frequency of hypoglycemia, volume overload, hypokalemia, LOS, and mortality during hospitalization between patients with DKA and ESRD and with preserved kidney function. Descriptive statistics were used to compare clinical and biochemical characteristics on admission and during hospitalization. The comparisons were made with the use of Wilcoxon tests for continuous variables and χ^2^ tests (or Fisher’s exact tests) for discrete variables. Multivariate logistic regression analysis was conducted for outcome hypoglycemia and volume overload, which was adjusted by age, sex, race, body mass index (and relevant comorbidities). A p value <0.05 was considered significant. All analyses were performed using SAS software V.9.4 (SAS Institute). The data are generally presented as mean±SD for continuous variables and n (%) for discrete variables.

## Results

Among 307 patients with DKA meeting the inclusion and exclusion criteria, a total of 68 (22.1%) patients had ESRD on chronic hemodialysis and 239 (77.9%) patients had preserved renal function (eGFR >60 mL/min). Baseline demographic characteristics and admission laboratory results are shown in table 1. There were no racial differences between groups, with the majority being African-American for both (71% vs 70%, p=0.88), but patients with ESRD and DKA were older (mean (±SD) age: 54.9±16.5 vs 35.6±14.1 years, p<0.001), more likely to be female (66% vs 51%, p=0.02), and to have government-sponsored (Medicare/Medicaid) insurance (74% vs 29%, p<0.01) compared with patients with DKA and preserved renal function.

**Table 1:** Clinical characteristics

Characteristic	ESRD n=68 (22.1%)	No ESRD n=239 (77.9%)	P value
Age (years)	54.9±16.5	35.6±14.1	<0.001
Sex, (n) %			0.02
Female	45 (66)	121 (51)	
Male	23 (34)	118 (49)	
Body mass index (kg/m^2^)	27.8±8.3	27.1±7.3	0.43
Race, (n) %			0.88
Caucasian	18 (26)	59 (25)	
African-American	48 (71)	168 (70)	
Others	2 (3)	12 (5)	
Ethnicity, (n) %			0.61
Hispanic	1 (1)	9 (4)	
Non-Hispanic	60 (88)	198 (83)	
Not reported	7 (10)	32 (13)	
Insurance, (n) %			<0.001
Commercial	15 (22)	88 (37)	
Government (Medicare/Medicaid)	50 (74)	68 (29)	
No insurance	3 (4)	82 (34)	
Comorbidities, n (%)			
Coronary artery disease	14 (21)	7 (3)	<0.001
Hyperlipidemia	22 (32)	34 (14)	<0.001
Chronic obstructive pulmonary disease	8 (12)	12 (5)	0.06
Peripheral vascular disease	6 (9)	1 (0.4)	<0.001
Acute pancreatitis	2 (3)	10 (4)	>0.99
Homelessness	0	9 (4)	0.22
Depression	10 (15)	27 (11)	0.53
Substance abuse	2 (3)	18 (8)	0.27

Data presented as mean±(SD) or count (percentage), unless otherwise indicated.

ESRD, end-stage renal disease.

Despite having a lower mean HbA1c on admission (9.6%±2.1 vs 12%±2.5, p<0.001), the ESRD cohort had higher admission mean BG (804.5±362.6 mg/dL vs 472.5±137.7 mg/dL, p<0.001), as well as higher mean BG during the first 48 hours of treatment compared with the control group. In addition, patients with ESRD presented with higher anion gap (23.6±7 vs 20.1±4.5, p=<0.001), serum osmolarity (304.4±19.5 vs 291.1±11.2, p=<0.001), but lower beta-hydroxybutyrate (4.5±3.3 vs 6.2±2.3 mmol/L, p=0.01) levels compared with patients with DKA and preserved kidney function.

**Figure 1:**
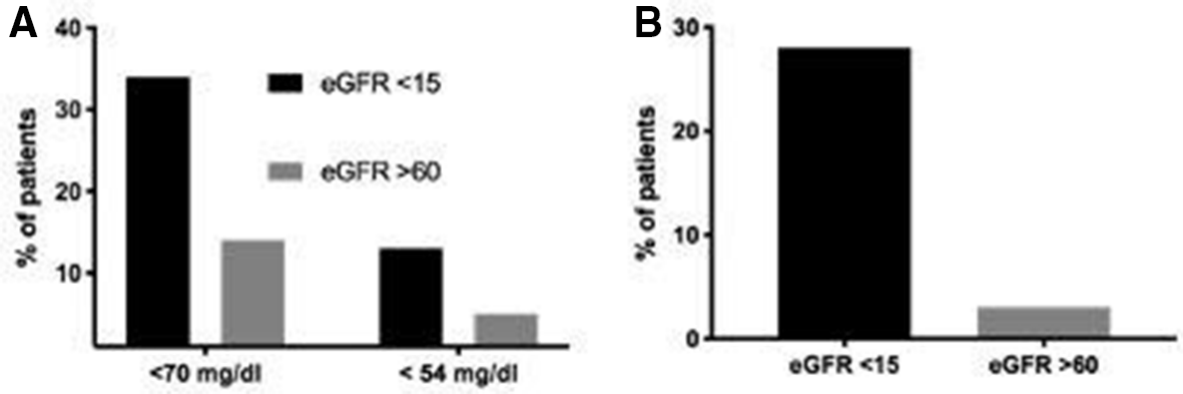
Proportion of patients developing (A) hypoglycemia and (B) volume overload after initiating therapy (all p<0.05). (A) Hypoglycemia <70 and <54 mg/dL. (B) Volume overload. eGFR, estimated glomerular filtration rate.

Metabolic parameters and response to medical therapy are shown in table 2. The time to correction of hyperglycemia (<250 mg/dL) was significantly longer in the ESRD group (8.4±2.6 hours vs 7.2±3.1 hours, p=0.03) compared with patients with preserved renal function. The rate of glucose reduction within the first 24 hours was higher in the ESRD group (−596.1±315.2 mg/dL vs −268.0±154.3 mg/dL, p=<0.001), and during the first 48 hours of therapy (−617.3±374.9 mg/dL vs −256.1±160.6 mg/dL, p=<0.001). The rates of hypoglycemia <70 mg/dL (34% vs 14%, p=<0.001) and <54 mg/dL (13% vs 5%, p=0.04) were twofold to threefold higher in the ESRD group compared with those with preserved renal function, but there were no differences in the rate of severe hypoglycemia <40 mg/dL between groups (figure 1).

**Table 2:** Metabolic parameters on admission and response to medical therapy

Metabolic parameters	ESRD	No ESRD	P value
HbA1c (%)	9.6±2.1	12±2.5	<0.001
Glucose (mg/dL)	804.5±362.6	472.5±137.7	<0.001
Sodium (mmol/L)	129.8±7.5	132.4±4.9	<0.001
Osmolality (mOsm/kg)	304.4±19.5	291.1±11.2	<0.001
Potassium (mmol/L)	5.8±1.4	4.6±0.9	<0.001
Anion gap (mEq/L)	23.6±7	20.1±4.5	<0.001
Bicarbonate (mEq/L)	12.6±4.0	13.0±3.9	0.47
Beta-hydroxybutyrate (mmol/L)	4.5±3.3	6.2±2.3	0.01
pH	7.1±0.2	7.2±0.2	0.42
**Response to therapy**			
Average BG days 1–10 (mg/dL)	268.9±86.3	255.5±70.3	0.35
Average BG day 1 (mg/dL)	629.4±304.6	364.2±114.7	<0.001
Average BG day 2 (mg/dL)	213.8±124.1	201.4±67.0	0.25
Average BG day 3 (mg/dL)	216.5±106.0	210.4±86.2	0.93
Average BG day 4 (mg/dL)	192.1±107.5	209.6±87.6	0.04
Average BG day 5 (mg/dL)	191.9±112.4	204.3±100.5	0.24
Average BG in first 48 hours	427.2±203.2	288.0±78.7	<0.001
Time to correction of hyperglycemia (BG <250 mg/dL), hours	8.4±2.6	7.2±3.1	0.03
Glucose reduction in first 24 hours (mg/dL)	−596.1±315.2	−268.0±154.3	<0.001
Glucose reduction in first 48 hours (mg/dL)	−617.3±374.9	−256.1±160.6	<0.001
Hypoglycemia <70 mg/dL, n (%)	23 (34)	34 (14)	0.002
Hypoglycemia <54 mg/dL, n (%)	9 (13)	13 (5)	0.04
Hypoglycemia <40 mg/dL, n (%)	2 (3)	4 (2)	0.62
Hypokalemia <3 mEq/L, n (%)	16 (24)	71 (30)	0.30
Hypokalemia <2.5 mEq/L	3 (4)	9 (4)	0.73

Data presented as mean±(SD) or count (percentage), unless otherwise indicated.

BG, blood glucose; ESRD, end-stage renal disease.

Patients with ESRD presented with higher potassium levels (5.8±1.4 mmol/L vs 4.6±0.9 mmol/L, p<0.001). During treatment, a similar number of patients experienced hypokalemia (<3 mmol/L), with no statistically significant differences (24% vs 30%, p=0.30) between groups.

Patients with DKA and ESRD experienced significantly higher rates of hospital complications including volume overload (28% vs 3%, p<0.001), myocardial infarction (10% vs 0.4%, p=<0.001), sepsis (22% vs 3%, p<0.001), deep venous thrombosis (7% vs 1%, p=0.01), and need for mechanical ventilation (24% vs 3%, p<0.001) compared with patients with DKA and preserved renal function, all p<0.001 (table 3). The all-cause hospital mortality was low in both groups (3% vs 0%, p=0.21), but was significantly higher in the ESRD cohort at 30 days (10% vs 1%, p<0.001) and at 1 year (18% vs 1%, p<0.001) compared with patients with preserved renal function. In addition, the LOS was significantly longer (median 7.0 days (IQR 4.0–12.5) vs median 3.0 days (IQR 2.0–4.0), p<0.001) and hospitalization costs were significantly higher in the ESRD cohort (median $50 844 (IQR $31 503–$77 730) vs median $14 252 (IQR $10 003–$23 886), p<0.001).

**Table 3:** Hospital complications and clinical outcomes

Complications, n (%)	ESRD	No ESRD	P value
Volume overload	19 (28)	7 (3)	<0.001
Mechanical ventilation	16 (24)	6 (3)	<0.001
Sepsis	15 (22)	8 (3)	<0.001
Myocardial infarction	7 (10)	1 (0.4)	<0.001
Rhabdomyolysis	3 (4)	0 (0)	0.01
Deep venous thrombosis	5 (7)	2 (1)	0.007
Pulmonary emboli	1 (1)	2 (1)	0.53
Mortality hospital	2 (3)	0 (0)	0.21
Mortality 30 days	7 (10)	2 (1)	<0.001
Mortality 365 days	12 (18)	3 (1)	<0.001
Length of stay, median days (Q1–Q3)	7.0 (4.0–12.5)	3.0 (2.0–4.0)	<0.001
Hospital costs, median US$ (Q1–Q3)	50 844 (31 503–77 730)	14 252 (10 003–23 886)	<0.001

ESRD, end-stage renal disease.

After adjusting for multiple variables including patient’s age, sex, race, and body mass index, we found that patients with DKA and ESRD have higher odds of hypoglycemia compared with patients with DKA and preserved renal function (OR 3.3, 95% CI 1.51 to 7.21, p=0.003). Similarly, in multivariate analysis adjusting for age, sex, race, body mass index, and history of coronary artery disease, we found that patients with ESRD have also an increased odds for volume overload (OR 4.22, 95% CI 1.37 to 13.05, p=0.01).

## Discussion

In this study, we analyzed the clinical characteristics and outcomes of patients hospitalized with DKA and ESRD on chronic maintenance hemodialysis. We report significant metabolic and clinical outcome differences in patients with DKA and ESRD compared with patients with preserved renal function. The ESRD group presented with twofold higher glucose levels and higher admission potassium concentration compared with those with preserved renal function. We also observed a twofold higher rate of hypoglycemia and a 10-fold higher rate of volume overload, compared with patients with preserved renal function. In addition, patients with ESRD had higher healthcare resource utilization, including longer LOS and hospitalization costs compared with patients with DKA and preserved renal function.

Diabetes is a leading cause of ESRD, with about half of patients requiring dialysis having a diagnosis of diabetes.^1–3^ There is limited high-quality evidence on the management and outcomes of DKA in patients with ESRD.^10^ The poor clinical outcome observed in these patients highlights the need for close metabolic monitoring and intensified treatment regimens to prevent the development of DKA, as well as the need for prospective studies to assess personalized management algorithms—including volume replacement and insulin therapy—to improve treatment-related complications and reduce healthcare costs in this high-risk population.

The mainstay of DKA management in patients with type 1 and type 2 diabetes includes the administration of intravenous fluids, insulin and electrolyte replacement therapy.^11^ For patients without cardiac or renal compromise, current guidelines recommend infusing isotonic saline at a rate of 15–20 mL/kg or 1–1.5 L during the first hour, with subsequent rate of 250–500 mL/hour.^11^ The higher rates of complications observed in our study suggest that treatment recommendations included in societies’ guidelines^11^ may not be applicable to patients with ESRD. First, we observed higher rates of volume overload suggesting that current recommendations for intravenous fluids resuscitation in DKA guidelines result in higher rates of fluid overload in the dialysis population. The main mechanism for the profound fluid and electrolyte depletion in DKA is osmotic diuresis, which is not present in dialysis patients with ESRD, increasing the risk of volume overload and pulmonary edema.^13^ Tzamaloukas *et al* 14 reported that the degree of hypertonicity and intracellular volume contraction is less profound in patients with ESRD compared with patients with normal renal function at similar hyperglycemia levels.

We suggest a modified approach for fluid replacement in patients with ESRD, preferring a small bolus of 250 mL initially with reassessment after each infusion—particularly if no hypotensive, or just insulin therapy without fluids bolus.^13^ Intensive fluid resuscitation during the initial management of DKA usually results in some degree of correction of hyperglycemia due to forced osmotic diuresis. However, hypertonicity in these patients is more a reflection of the degree of hyperglycemia and not due to osmotic diuresis, as previously discussed.^13^ It has been proposed that adequate insulin administration alone, with limited or without intravenous fluid resuscitation, may resolve the metabolic disturbances without complications.^13^ Current guidelines recommend an insulin infusion with a glucose correction rate of 50–75 mg/dL/hour. Using continuous glucose monitoring on patients with diabetes, Kazempour-Ardebili *et al* 15 showed lower mean glucose levels by −36 to −190 mg/dL during dialysis days compared with days off dialysis; and Sobngwi *et al* 16 showed that total daily insulin needs can decrease by ~25% on hemodialysis days. Hence, we suggest a careful and slower rate of hyperglycemia correction, considering the glucose-lowering effect of dialysis and/or potentially avoiding the use of intravenous insulin bolus on starting the insulin infusion, which may decrease the risk of hypoglycemia. However, future studies are needed to determine the best hydration regimen to manage patients with DKA and ESRD to minimize iatrogenic fluid overload.

Potassium concentration is maintained in the body by balancing the intake with the excretion and the transcellular shifts of potassium. Approximately 75% of patients admitted with DKA present with elevated serum potassium levels on admission owing to the shift of intracellular potassium to the extracellular compartment in the setting of hypertonicity, insulin deficiency and acidosis.^5 17^ As expected, patients with ESRD in this study presented with higher potassium levels due to diminished potassium excretion by the kidneys.^17 18^ During insulin treatment, potassium concentration rapidly decreases due to intracellular shifting of potassium, mediated by insulin stimulation of Na^+^-H^+^ transporter, promoting the entry of Na^+^, then activation of the Na^+^-K^+^ ATPase, resulting in influx of potassium^19^; as well as increased kaliuresis, thought to be mediated by an aldosterone-like effect of insulin.^20^ In recent studies in patients with ESRD, the prevalence of hyperkalemia ≥5 mmol/L was reported between 14% and 20%, and the prevalence of hypokalemia ≤4 mmol/L was between 12% and 18%,^17^ with both conditions associated with increased mortality.^21^ Patients with DKA and ESRD presented with higher potassium concentration compared with patients with preserved renal function. In agreement with previous reports, insulin administration was the only treatment required for correction of hyperkalemia.^18 21^ Of interest, we observed no differences in the rate of hypokalemia between groups. These results indicate the need for a modified potassium administration approach in the management of DKA in patients with ESRD, as well as close follow-up of potassium levels on admission and during insulin treatment.

We acknowledge several limitations in our analysis including a relatively small number of patients with ESRD presenting with DKA; however, this is the largest cohort study of patients with ESRD admitted with DKA—an uncommon scenario.^10^ The retrospective nature of the study and the use of ICD-9 codes may limit the ability to accurately differentiate between type 1 and type 2 diabetes, duration of diabetes and precipitant causes. Nonetheless, despite using ICD-9/10 codes, we were able to confirm all cases of DKA and ESRD based on biochemical data on admission, which have been a limitation of previous reports. In addition, although a DKA treatment protocol is available for use in the emergency department and in intensive care unit and step-down units, the current protocols are not designed to adjust for changes in insulin, fluid and electrolyte administration in patients with diabetes and ESRD on hemodialysis. We also recognized the limited value of HbA1c in the setting of ESRD.

In conclusion, we report significant metabolic and clinical outcome differences between patients with DKA and ESRD compared with patients with preserved renal function. Patients with DKA and ESRD on hemodialysis have higher rates of complications, increased resource utilization and hospitalization costs than patients with DKA and preserved renal function. We observed a greater number of patients with DKA and ESRD developing volume overload and hypoglycemia during insulin treatment compared with patients with preserved renal function. These results indicate that a personalized and careful approach to insulin and fluid replacement and correction of hyperglycemia is needed to improve outcome and prevent iatrogenic complications, volume overload and hypoglycemia.
