# Supplementary material for: Clinical characteristics and outcomes of patients with end-stage renal disease hospitalized with diabetes ketoacidosis
Source: BMJ Open Diabetes Res Care. 2020 Feb 27;8(1):e000763. doi: 10.1136/bmjdrc-2019-000763 (PMC7050364; doi:10.1136/bmjdrc-2019-000763)
Supplement: Supplementary data [file bmjdrc-2019-000763supp001.pdf]

ICD-9 codes used for cohort identification

a) Diabetes ketoacidosis: 250.10 (type 2 or unspecified, not uncontrolled), 250.11 (type 1, or juvenile), 250.12 (type 2 or unspecified, uncontrolled), 250.13 (type 1, uncontrolled), and 250.3 (diabetic coma, diabetic hypoglycemic coma, insulin coma, but only if admission blood glucose > 600 mg/dl)

b) End-stage renal disease on chronic hemodialysis: 585.6 (CKD stage V requiring dialysis), V45.1, V45.11, V56.0 (hemodialysis)

Laboratory diagnostic criteria used to confirm cases of diabetes ketoacidosis

a) BG > 250 mg/dl, bicarbonate ≤18 mEq/L on admission, or measured beta-hydroxybutyrate > 3 + pH < 7.3 on admission, positive ketones + pH < 7.3.

a) bicarbonate ≤18 mEq/L on presentation plus osmolality ≥300 mOsm/kg, or measured Betahydroxybutyrate > 3 + HHS features (BG > 500 + osmolality >300)
